# Historias de sugestión y magnetismo

**DOI:** 10.1590/S0104-59702024000100037

**Published:** 2024-07-26

**Authors:** María Silvia Di Liscia

**Affiliations:** i Instituto de Estudios Históricos y Sociales de La Pampa/Universidad Nacional de La Pampa. Santa Rosa – La Pampa – Argentina silviadiliscia@gmail.com

**Figure:**
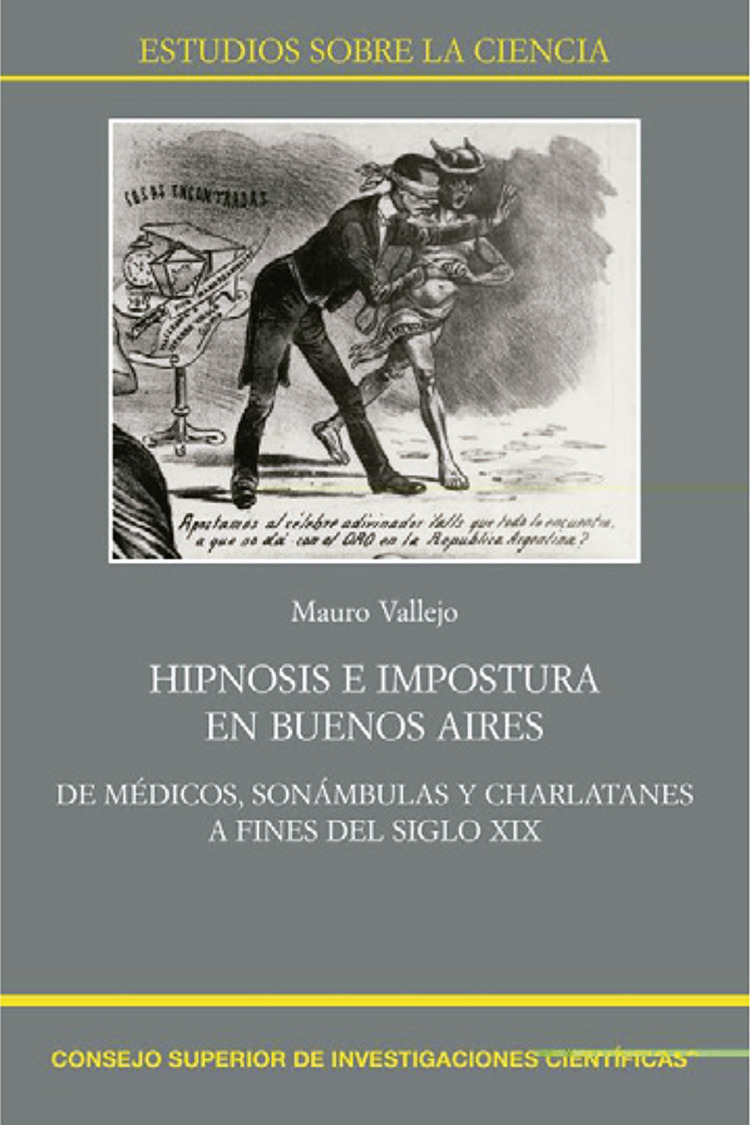
VALLEJO, Mauro. *Hipnosis e impostura en Buenos Aires: de médicos, sonámbulas y charlatanes a fines del siglo XIX*. Madrid: Consejo Superior de Investigaciones Científicas, 2021. 348p.

Las enormes posibilidades técnicas de la medicina, de las cuales somos bien conscientes en la actualidad, permitieron, en muchos casos, evitar el pasado. Pero es imposible, y mucho menos para cualquier historiador que cultive la disciplina tanto en nuevos como añejos formatos, olvidar que éstas hicieron a las sociedades humanas más sanas y viejas. También, a consecuencia del alargamiento de vida y de la imposibilidad de resolver la vejez con todas sus implicancias, más desiguales y quizás menos felices (Porter, 1997).

Para muchas lecturas sesgadas, que sugieren una curva ascendente y gloriosa del progreso médico, y no ese pesimismo que estamos vislumbrando, los tiempos pretéritos alumbraron errores y fantasías, aupados en la superstición de gran parte de la sociedad, deseosa de resolver a toda costa sus problemas de salud. Justamente, el magnetismo, parte de una cultura técnica hoy pseudocientífica, nació a finales del siglo XVIII, aprovechando las ventajas del impacto de la electricidad y de sus casi mágicas aplicaciones, y se extendió por Europa y América con un reguero de certezas, para vencer todas las dolencias humanas. El hipnotismo también asumió esa fascinación, ya que unía saberes probadamente reconocidos con aquellos inexplorados: la dominación de las mentes a través de mecanismos desconocidos abría una puerta al control de los cuerpos (y qué hay más de fascinante, para la mentalidad decimonónica, que la esclavitud). Las capacidades de decisión individual desaparecían, borradas no por el látigo o la norma, sino por invisibles lazos mentales.

Este libro narra convincentemente, a través de uno de esos profesionales de la hipnosis, la fascinante historia de esta y otras prácticas, en el borde mismo (casi diríamos, en el precipicio) de la medicina aceptada oficialmente. Con una prosa atrayente, Vallejo madura argumentos con preguntas contundentes: ¿Qué es la medicalización? ¿Cuáles son sus elementos básicos? ¿Cómo se produce ese encuentro entre el Estado y sus oficiantes? Si unos constituyen la esfera de lo permitido y otros de lo prohibido, ¿qué hay entonces en medio?

La obra barrunta sobre justamente estos facultativos que se encuentran en las bisagras “grises”, navegan, a veces exitosamente, entre las aguas confusas de una medicina – que, a pesar de sus éxitos diagnósticos, no consigue total eficacia en la cura – y pescan con éxito sus clientes, en el mar impreciso pero riquísimo de las prácticas “ilegales”. De esta manera se acumulan, generosamente aceptadas por diferentes estratos sociales, las posibles curas a diferentes males mentales (neurosis, clorosis, neurastenia, y tanto más), a su vez despreciadas y combatidas por academias, cátedras y otras instituciones reguladas por la medicina oficial.

La implicancia médica, sin embargo, no es el único aliciente, antes bien, es la mercantilización de las posibilidades de uso de estas técnicas que orillan o disienten abiertamente con las experiencias ya consolidadas. Recordemos que desde mediados del siglo XIX, tanto a partir del estudio anatomo-clínico como bacteriológico, el conocimiento del cuerpo humano y de sus disfunciones avanzó a pasos agigantados, junto a la descripción de las causales de enfermedades. Esa verdadera revolución que dio por tierra el sistema humoral y a la vez, las explicaciones sobre emanaciones miasmáticas, sin embargo, no fue tan eficaz a la hora de curar o consolar a los enfermos mentales.

En el margen, surgió una legión de personas dedicadas a resolver esas situaciones, con el estímulo económico, la capacidad y el conocimiento y también, por qué no, cierta abnegación frente al dolor ajeno. Ninguno de esos factores se olvida en *Hipnosis e impostura,* que a través de seis capítulos hilvana coherente y atractivamente la vida de un profesional, representante de una legión: Alberto Díaz de la Quintana y Sánchez Remón. Médico español, aunque sin reconocimiento legal en Buenos Aires, sumó clínicas y demostró teatralmente, en escenarios cuidadosamente preparados, la potestad de sus prácticas en performances con sonámbulas adiestradas y artificios técnicos. Pero no como emanados de encantamientos mágicos, sino, y esto es lo interesante, buscando el reconocimiento legal y científico entre sus pares profesionales.

La precisa narración de Vallejo (2021a, p.28) nos conduce a través de este personaje “publicista, inventor, dramaturgo y poeta” por los intersticios de un mundo realmente casi inexplorado, el de los denominados charlatanes por los médicos, pero refundados por la comunidad como curadores bienhechores, o incluso, autodefinidos como verdaderos científicos. El texto tiene muchos aciertos, entre ellos el uso de una erudición ciertamente implacable: a través de numerosas fuentes y una variada documentación obtenida tanto en Argentina como en España, se demuestra de manera convincente la diversidad de la oferta curativa en una ciudad, como Buenos Aires, abierta a las novedades europeas. Es esa urbe donde se escuchaban los acentos de miles y miles de recién llegados, un espacio favorecido para, por un lado, las “nuevas” patologías mentales, frutos de esos tiempos desosegados e inciertos, y, por otro, una batería de también novedosas curas.

Como también en otra de sus obras (Vallejo, 2021b), el autor focaliza en las enormes posibilidades abiertas por el mercado terapéutico porteño. Se abre entonces una comunidad ansiosa y sufriente, incluso (y esta es la ironía) de dolencias antes desconocidas, o creadas por los mismos médicos, que puede pagar por baños eléctricos o píldoras sanadoras de composición secreta. No importan los certificados que acrediten los conocimientos de los facultativos, sino el “boca a boca” y la capacidad de relacionarse y publicitarse eficazmente.

Sin duda, esta capacidad de utilizar diversos recursos, sin excluir los teatrales, nos permiten acercarnos a la diversidad de la oferta médica, ya estuviese aceptada, al margen u opuesta a las regulaciones. Otro de los aciertos del libro es reflexionar sobre quiénes son estos personajes contradictorios que, sin carecer de los títulos médicos, navegan entre las aguas de una cultura sanitaria indefinida. Dice Vallejo (2021a, p.328): “Nos hemos habituado a medir el accionar de los médicos de fines de siglo con categorías de análisis que suponen siempre una operatoria de relevo o suplencia” como charlatanes o aventureros. Y sigue afirmando: “Debemos enfrentar el desafío de tomar desde un punto de vista positivo los pilares de algo que aún no se había constituido, o de un orden que podía ser violado” (p.328). La clave estaría, entonces, en considerar la curación de los cuerpos enfermos como un proceso terminado, cuya llave estaba en manos de un circuito oficialmente constituido en lo que quizás, pomposamente, hemos llamado “medicina oficial”. Es entonces esa indeterminación en el juego la que presenta el libro, llamando desde el siglo XIX a reflexionar sobre nuestros sistemas médicos, legales y no tanto, a la luz del presente.
